# The Level of Knowledge and Attitudes Toward Dementia Among Senior Medical Students in Recife, Brazil

**DOI:** 10.7759/cureus.45294

**Published:** 2023-09-15

**Authors:** Ivo Wandark Filho, Zenildo Ernesto Ferraz Segundo, Arthur Felipe Cordeiro Fraga, Maria Letícia Carnielli Tebet, Eduardo Ribas Izidro Gomes, Eduardo Jorge Abrantes da Fonte

**Affiliations:** 1 School of Medicine, Faculdade Pernambucana de Saúde (FPS), Recife, BRA; 2 School of Medicine, Pontifícia Universidade Católica do Paraná, Curitiba, BRA; 3 Department of Geriatrics, Instituto de Medicina Integral Professor Fernando Figueira (IMIP), Recife, BRA

**Keywords:** cognitive changes, brazil, attitudes, knowledge, students, dementia

## Abstract

Introduction: Aging is no longer a phenomenon for society; it has become a reality in all countries, leading to a notable increase in the prevalence of dementia, a common condition among the elderly population. This situation highlights the importance of adequately preparing future healthcare professionals with the necessary knowledge and attitudes to effectively care for dementia patients.

Objective: This study aims to describe the knowledge and attitudes toward dementia among fifth- and sixth-year medical students at a prestigious medical school in Recife, Brazil.

Materials and methods: A descriptive, analytical cross-sectional study was conducted in which participants answered questionnaires related to epidemiological and educational data of the involved students, the assessment of the sample’s knowledge regarding dementia, and addressing attitude toward a patient with dementia. Data collection took place online, targeting fifth- and sixth-year medical students at the Faculdade Pernambucana de Saúde (FPS).

Results: A total of 111 students participated in the study, with a majority of females (73.9%), most of them in the fifth year of medical school (79.3%). While the majority of the students received training during their undergraduate studies on cognitive changes related to dementia (58.6%), this knowledge was mostly theoretical (64%), and only a few students took extracurricular courses on the subject (7.2%). Regarding the questionnaire evaluating students’ knowledge, the overall mean was 6.69 points (on a scale of 0-14). Notably, there was no significant difference in correct answers among the tested areas of epidemiology, diagnosis, and management, with percentages of correct answers of 49.8%, 45.27%, and 52.53%, respectively. As for their attitudes toward dementia, the majority of students responded in a manner consistent with current literature and best practices for managing patients with functional dependence and cognitive changes.

Conclusion: The results indicate that despite the notable rise in dementia cases across the world, the study revealed that the participants lacked essential knowledge about dementia. However, most of them demonstrated attitudes aligned with the best practices for managing dementia patients and their families. These data may suggest the need for greater attention in the teaching-learning process on the part of the medical school, as well as the promotion of extracurricular activities on this topic, in addition to enhancing the promotion of practical activities in geriatrics.

## Introduction

Aging has ceased to be merely a phenomenon for society and has already become a part of reality in all countries. By 2050, it is estimated that the proportion of the world’s population over 60 years old will nearly double. In Brazil, there are an estimated 17.6 million elderly individuals, with 75.3% of them relying on the services of the Public Unified Brazilian Health System, Sistema Único de Saúde (SUS). Aging is a natural process, associated with a progressive functional decline in individuals, senescence, which generally does not cause any problems. However, under conditions of overload, such as diseases, accidents, and emotional stress, it can result in a pathological condition that requires proper care, senility. This new event can bring a series of challenges for healthcare systems [1,2].

It is known that nerve cells are affected by various intrinsic and extrinsic factors during the aging process, which exerts harmful effects over time. Throughout the life of an aging individual, discrete signs of functional deficiencies may appear, sometimes without compromising personal, executive, and managerial activities, which can be characterized as healthy aging [2].

Senility, or pathological aging, occurs when there is more severe damage, leading to significant functional impairments, especially related to the individual’s cognitive ability, affecting memory, reasoning, praxic and gnostic functions, and communication, significantly compromising their autonomy and independence. Dementia mainly affects the elderly through progressive and irreversible neurodegenerative processes, potentially having disastrous consequences for each individual and directly affecting the family and society [3-6].

The prevalence of dementia can vary among different regions worldwide. It is estimated that 24.3 million people have dementia syndrome, with an incidence of 4.6 million new cases each year. Studies indicate that dementia cases are expected to double every 20 years, resulting in an increase to 81.1 million cases by 2040. In the United States, in 2015, 5.3 million people were diagnosed with dementia, primarily Alzheimer’s disease (AD) [7,8]. In Brazil, the prevalence has increased considerably in the last 30 years due to the rapid aging of the population [9].

Recent studies show the prevalences of each subtype of dementia: Alzheimer’s disease (35.4%), vascular dementia (21.2%), mixed dementia (13.3%), and other causes of dementia (30.1%). The frequency of dementia may vary depending on the age group. Alzheimer’s disease (AD) is less frequent in individuals with early-onset dementia (<65 years) compared to those over 65, where it remains the most common etiology. On the other hand, frontotemporal dementia (FTD) is significantly more common in the younger age group compared to individuals over 65 [1].

Many healthcare professionals underdiagnosed cognitive deficits in elderly individuals, especially those in the early stages of dementia. In some cases, it may be mistaken for normal aging, as functional performance is still relatively preserved. This difficulty in identifying the characteristics of a patient with pathological cognition has been studied in various countries, including Brazil [9,10].

Evidence shows that it is unclear whether early diagnosis of dementia can help improve a patient’s performance, despite the Brazilian medical society recommending tests to assess cognitive function as part of comprehensive geriatric evaluation (CGA), facilitating the planning and care process by a multidisciplinary team in the comprehensive care of the elderly [9,11].

It is known that the vast majority of the elderly population in Brazil is served by SUS, which largely consists of general practitioners, including a proportion of recent graduates who play a crucial role in recognizing, managing, and treating diseases with high prevalence in the country, especially dementia [6,9,12]. However, many general practitioners, including recent graduates, neglect cognitive impairment in the elderly due to a lack of knowledge, as it is often mistaken for the natural physiological aging process of the elderly [6,13-16]. This raises the following question: with the increasing cases of dementia in the Brazilian population, do recent medical graduates have sufficient knowledge to recognize and manage patients with dementia?

Thus, this study aims to describe the knowledge and attitudes about dementia among fifth- and sixth-year medical students at a renowned private medical school in Recife, Brazil.

This article was previously presented as a poster at the XIII Faculdade Pernambucana de Saúde (FPS) Student Conference on October 28, 2022.

## Materials and methods

A descriptive, analytical cross-sectional study was conducted, where questionnaires with specific questions were administered to the participants. The study was conducted online for students of the Faculdade Pernambucana de Saúde (FPS) in Recife, Brazil.

The study sample consisted of fifth- and sixth-year (last two years of medical school in Brazil) medical students from April to June 2022, with a total of 111 participants. The inclusion criteria were students enrolled in the fifth and sixth year of the medical course at the Faculdade Pernambucana de Saúde (FPS). The exclusion criteria were students who did not complete the questionnaire.

The first questionnaire pertains to the collection of epidemiological and educational data from the involved students. The second questionnaire involves assessing the sample’s knowledge regarding dementia, encompassing questions related to epidemiology, diagnosis, and dementia management. The final questionnaire addresses attitudes toward a patient with dementia [7,9,11,13]. These last two questionnaires were developed by researchers in the United Kingdom (UK) and administered by them in a study aimed at evaluating the knowledge and attitudes of general practitioners in the UK [13]. Subsequently, these questionnaires were translated, culturally adapted to the Brazilian context, and replicated in a study involving final-year medical students from two universities in São Paulo, Brazil [7,9,11].

The research team provided a link to all participants for the questionnaires, conducted online through Google Forms (Google, Inc., Mountain View, CA, USA). Data collection took place from April to June 2022.

For statistical analysis and data management, Google Forms was used, a web application that allows for meta-analysis to consult, search, sort, filter, and calculate data obtained through questionnaires completed by participants.

This project was approved by the Ethics Committee of FPS (CAAE 53748821.0.0000.5569) and followed the guidelines and regulatory norms for research involving human subjects according to resolution 510/16 of the Brazilian National Health Council.

The project offers the benefit of better understanding the knowledge and attitudes of fifth- and sixth-year students on the topic of dementia. Participants did not suffer any physical harm or aggravation, and their participation in this project did not bring significant changes to their routine, as students took about 10 minutes to answer the questionnaires. There were no personal expenses for the participants in the research, and their participation was not remunerated. The individual results of each participant will not be disclosed.

## Results

Table 1 shows the epidemiological and educational data from the participants. The study involved a total of 111 students, with a predominance of female participants (73.9%). Additionally, most of the students were in the fifth year of medical school (79.3%). Regarding the question of whether they received training on cognitive changes in dementia, 58.6% responded affirmatively. However, this knowledge was predominantly theoretical (64%). A small proportion of students reported taking an extracurricular course on the subject (7.2%).

**Table 1:** General aspects of the medical students during their training FPS: Faculdade Pernambucana de Saúde

Items	FPS students
Sex	
Male	29 (26.1%)
Female	82 (73.9%)
Year	
5th Year	88 (79.3%)
6th Year	23 (20.7%)
Had good training on cognitive changes?	
Yes	65 (58.6%)
No	35 (31.5%)
Can’t remember	11 (9.9%)
If yes, the training on cognitive changes was:	
Theoretical only	71 (64%)
Theoretical and practical	30 (27%)
Can’t remember	10 (9%)
Took any extracurricular course on the subject?	
Yes	8 (7.2%)
No	103 (92.8%)

The data analysis in Table 2 focuses on questions related to knowledge about dementia. In questions related to epidemiology, there was a low percentage of correct answers regarding the prevalence of dementia (33.3% and 34.2%). However, most students answered correctly regarding risk factors related to dementia (82%). As for questions about the diagnosis of dementia, satisfactory performance was observed in four out of eight questions, where students achieved an accuracy rate of over 60%. However, questions evaluating definitive diagnosis, differential diagnosis, and symptomatology showed low performance (30.6%, 15.3%, and 15.3%, respectively).

**Table 2:** Correct answers in the knowledge questionnaire ABRAZ: Brazilian Alzheimer’s Association

Questions	Number of correct answers (%)
Epidemiology	
1. A general practitioner with a list of 1,000 people aged 60 or older should expect to have approximately how many people with dementia on this list?	37 (33.3%)
2. From the age of 65 years old, what is the prevalence of dementia?	38 (34.2%)
3. Which of the following is a risk factor for the development of Alzheimer’s disease?	91 (82%)
Diagnosis	
4. All of the following are potentially treatable etiologies of dementia except:	86 (77.5%)
5. Why should a patient suspected of having dementia be evaluated as soon as possible?	79 (71.2%)
6. Which of the following procedures is necessary to definitively confirm that the symptoms are caused by dementia?	34 (30.6%)
7. Which of the following is not a necessary part of the initial evaluation of a patient suspected of dementia?	89 (80.2%)
8. Which of the following can resemble dementia?	17 (15.3%)
9. When a patient presents a sudden onset of confusion, disorientation, and an inability to maintain attention, this picture is most compatible with the diagnosis of:	17 (15.3%)
10. Which of the following options is almost always present in dementia?	13 (11.7%)
11. Which of the following clinical findings best differentiates vascular dementia from Alzheimer’s disease?	67 (60.4%)
Management	
12. The effect of anti-dementia medications acts on:	84 (75.7%)
13. Which statement is true about the treatment of patients with dementia who are depressed?	45 (40.5%)
14. ABRAZ is the Brazilian association that provides information to patients and caregivers for what purpose?	46 (41.4%)

Regarding questions about dementia management, most students answered correctly the question about the effects of medications used in treatment (75.7%). However, they encountered difficulties in identifying the treatment of dementia in depressed patients and in understanding the purpose of the Brazilian Alzheimer’s Association (ABRAZ) in providing information to patients and caregivers, with an accuracy rate of 40.5% and 41.4%, respectively.

Table 3 presents the attitudes of medical students regarding dementia. Most students agreed that there is much to be done to improve the quality of life of patients with dementia and their caregivers (99.1%). Moreover, the majority disagreed with using euphemisms when approaching these patients (75.6%). It was also observed that most students agree that dementia is best diagnosed in specialized services (74.7%).

**Table 3:** Answers to the attitudes questionnaire

Statements	Strongly agree (%)	Agree (%)	Neither agree nor disagree (%)	Disagree (%)	Strongly disagree (%)
1. Much can be done to improve the quality of life for caregivers of people with dementia.	97 (87.4%)	13 (11.7%)	1 (0.9%)	0 (0%)	0 (0%)
2. Families prefer to be informed about their relative’s dementia as soon as possible.	54 (48.6%)	38 (34.2%)	16 (14.4%)	3 (2.7%)	0 (0%)
3. Much can be done to improve the quality of life for people with dementia.	94 (84.7%)	16 (14.4%)	1 (0.9%)	0 (0%)	0 (0%)
4. Providing a diagnosis is generally more helpful than harmful.	56 (50.5%)	37 (33.3%)	13 (11.7%)	3 (2.7%)	2 (1.8%)
5. Dementia is best diagnosed in specialized services.	39 (35.1%)	44 (39.6%)	20 (18%)	5 (4.5%)	3 (2.7%)
6. Patients with dementia can exhaust resources with little positive outcome.	20 (18%)	25 (22.5%)	33 (29.7%)	26 (23.4%)	7 (6.3%)
7. It is better to talk to the patient using euphemisms.	4 (3.6%)	5 (4.5%)	18 (16.2%)	45 (40.5%)	39 (36.1%)
8. Treating dementia is usually more frustrating than rewarding.	11 (9.9%)	18 (16.2%)	28 (25.2%)	41 (36.9%)	13 (11.7%)
9. It’s not worth directing families to specialized services when they don’t want to use them.	6 (5.4%)	11 (9.9%)	22 (19.8%)	47 (42.4%)	25 (22.5%)
10. The primary care team has a very limited role in the care of people with dementia.	6 (5.4%)	12 (10.8%)	6 (5.4%)	33 (29.7%)	54 (48.6%)

## Discussion

The study evaluated that the majority of participants were female (73.9%), with theoretical training during their undergraduate studies (64%), but without engaging in extracurricular activities related to dementia (92.8%). The predominance of female participants can be attributed to the increased integration of females into medical courses nowadays [17]. The lack of practical training and extracurricular activities is a matter of concern since it could lead to evident improvements in students’ perceptions and knowledge [18].

Regarding the questionnaire that evaluated students’ knowledge during their undergraduate studies, an average of 6.69 points (on a scale of 0-14) was recorded. This suggests that the knowledge acquired in managing a patient with dementia might have been insufficient. This finding is consistent with another Brazilian study on the same topic conducted in São Paulo, where students obtained an average of 6.9 points. This demonstrates that the educational issue is likely a national problem and calls for further studies to determine if this gap applies to other regions in Brazil [7].

In terms of the rates in different tested areas, such as epidemiology, diagnosis, and management, our study shows accuracy rates of 49.8%, 45.27%, and 52.53%, respectively. These numbers are similar to those found in the study conducted by Jacinto et al. among residents from a university hospital in São Paulo, which showed rates of 54.9%, 58.7%, and 76.3% [11]. The difference in the management rate can possibly be explained by the fact that the study population was predominantly composed (51.9%) of doctors who had graduated more than a year before the study and possibly had professional experiences before entering residency. This could also explain the percentages found in a study from the United Kingdom that evaluated the same topic among general practitioners and found percentages of 48%, 74%, and 73% on the same subscales [13].

Our study also demonstrated that a high percentage of the students showed deficits regarding the knowledge of the epidemiology of the disease, considering the first two questions of this section of the survey. This is concerning in a public health system such as the Brazilian one because it shows that the future general practitioners can under- or overestimate the number of patients with the disease, not providing proper care for those who suffer from the condition or increasing the need for further workup in the context of a system with limited resources [19].

When evaluated in relation to their attitudes toward dementia, the majority of students responded consistently with current literature and best recommendations for the management of patients with functional dependence and cognitive impairments, for example, in questions regarding the need to avoid using euphemisms (question 7) or questions about the potential to enhance the quality of life for family members, caregivers, and patients themselves. However, a significant number of students still believe in the necessity of specialized services to provide accurate diagnoses, which are not feasible within the Brazilian public health system. They also perceive limitations in primary care for managing dementia, possibly due to insecurity and a lack of theoretical-practical knowledge in geriatrics and dementia conditions [12-16,20,21].

Nonetheless, it is important to highlight the study’s limitations. The results were derived from students attending a private medical school in northeastern Brazil and had a limited sample, especially from the final year. Furthermore, concerning the survey, question 12, which addresses the effects of anti-dementia medications, includes the correct answer that these drugs used in dementia management might temporarily interrupt the disease in some cases. However, the main references indicate that these medications lead to symptomatic improvement without altering the disease’s course [1,22,23]. Moreover, question 9, which had an accuracy rate of only 15.3%, describes in its statement symptoms present in acute confusional state, the option most chosen by students, but the correct answer according to the official questionnaire is vascular dementia, making the question ambiguous and limiting its ability to evaluate knowledge without bias [24,25].

## Conclusions

The results indicate that despite the notable rise in dementia cases across the world, the study revealed that the participants lacked essential knowledge about the disease. However, a majority of them demonstrated attitudes aligned with the best practices for managing dementia patients and their families. It is worth noting that these participants acknowledged the importance of specialized services for diagnosing this condition, yet they do not perceive primary care as having a restricted role in dementia care.

These data may suggest the need for greater attention in the teaching-learning process on the part of the medical school, as well as the promotion of extracurricular activities on this topic, in addition to enhancing the promotion of practical activities in geriatrics, given that the portion of students who claimed to have received training on the topic stated that the training was solely theoretical.

## Appendices

Table 4 shows the knowledge questionnaire used in the present study.

**Table 4:** Knowledge questionnaire ABRAZ: Brazilian Alzheimer’s Association Answers to the knowledge questionnaire: 1. D, 2. A, 3. B, 4. C, 5. C, 6. B, 7. D, 8. A, 9. D, 10. B, 11. C, 12. B, 13. B, 14. A

Questions	Options
1. A general practitioner with a list of 1,000 people aged 60 or older should expect to have approximately how many people with dementia on this list?	A. 10
	B. 500
	C. 200
	D. 70
	E. I don’t know
2. From the age of 65 years old, what is the prevalence of dementia?	A. Doubles every 5 years
	B. Doubles every 10 years
	C. Doubles every 15 years
	D. Doubles every 20 years
	E. I don’t know
3. Which of the following is a risk factor for the development of Alzheimer’s disease?	A. Arterial hardening
	B. Age
	C. Nutritional deficiencies
	D. Exposure to aluminum
	E. I don’t know
4. All of the following are potentially treatable etiologies of dementia except:	A. Hypothyroidism
	B. Normal-pressure hydrocephalus
	C. Creutzfeldt-Jakob disease
	D. Vitamin D deficiency
	E. I don’t know
5. Why should a patient suspected of having dementia be evaluated as soon as possible?	A. Immediate treatment for dementia can prevent worsening of symptoms.
	B. Immediate treatment for dementia can reverse the symptoms.
	C. It is important to rule out and treat reversible disorders.
	D. It is best to institutionalize a patient with dementia early in the disease.
	E. I don’t know.
6. Which of the following procedures is necessary to definitively confirm that the symptoms are caused by dementia?	A. Mini-Mental State Examination
	B. Post-mortem examination
	C. Brain tomography
	D. Blood test
	E. I don’t know
7. Which of the following is not a necessary part of the initial evaluation of a patient suspected of dementia?	A. Thyroid function test
	B. Serum electrolytes
	C. Vitamin B and folic acid levels
	D. Protein electrophoresis
	E. I don’t know
8. Which of the following can resemble dementia?	A. Depression
	B. Acute confusional state
	C. Stroke
	D. All of the above
	E. I don’t know
9. When a patient presents a sudden onset of confusion, disorientation, and an inability to maintain attention, this picture is most compatible with the diagnosis of:	A. Alzheimer’s disease
	B. Acute confusional state
	C. Major depression
	D. Vascular dementia
	E. I don’t know
10. Which of the following options is almost always present in dementia?	A. Memory loss
	B. Memory loss and incontinence
	C. Memory loss, incontinence, and hallucinations
	D. None of the above
	E. I don’t know
11. Which of the following clinical findings best differentiates vascular dementia from Alzheimer’s disease?	A. Word-finding difficulties
	B. Loss of immediate visual memory (2 minutes)
	C. Stepwise disease development (plateaus with stabilization, interspersed with sudden decline)
	D. Presence of depression
	E. I don’t know
12. The effect of anti-dementia medications acts on:	A. Temporarily stop the disease in all cases
	B. Temporarily stop the disease in some cases
	C. Temporarily stops the disease in some cases but often causes liver damage
	D. Permanently stop the disease in some cases
	E. I don’t know
13. Which statement is true about the treatment of patients with dementia who are depressed?	A. It is generally useless to treat them for depression, as feelings of sadness and inadequacy are part of the disease.
	B. Depression treatments can be effective in relieving depressive symptoms.
	C. Antidepressant medications should not be prescribed.
	D. The correct medication can alleviate symptoms of depression and prevent future intellectual decline.
	E. I don’t know.
14. ABRAZ is the Brazilian association that provides information to patients and caregivers for what purpose?	A. To help people better understand the disease so they can cope with symptoms and treatments more effectively
	B. Free outpatient medical care
	C. Recruiting people with dementia for research
	D. All of the above
	E. I don’t know
